# Haematological profile of malaria patients with *G6PD* and *PKLR* variants (erythrocytic enzymopathies): a cross-sectional study in Thailand

**DOI:** 10.1186/s12936-022-04267-7

**Published:** 2022-08-30

**Authors:** Punchalee Mungkalasut, Patcharakorn Kiatamornrak, Watcharapong Jugnam-Ang, Srivicha Krudsood, Poonlarp Cheepsunthorn, Chalisa Louicharoen Cheepsunthorn

**Affiliations:** 1 Interdisciplinary Programme of Biomedical Sciences, Graduate School, Chulalongkorn University, Bangkok, Thailand; 2 Medical Biochemistry Programme, Department of Biochemistry, Faculty of Medicine, Chulalongkorn University, Bangkok, Thailand; 3 Department of Tropical Hygiene and Clinical Malaria Research Unit, Faculty of Tropical Medicine, Mahidol University, Bangkok, Thailand; 4 Department of Anatomy, Faculty of Medicine, Chulalongkorn University, Bangkok, Thailand; 5 Department of Biochemistry, Faculty of Medicine, Chulalongkorn University, 1873 Rama 4 Rd., Pathumwan, Bangkok, 10330 Thailand

**Keywords:** G6PD deficiency, Pyruvate kinase, Erythrocyte enzymopathy, *G6PD* Mahidol, Thailand, Southeast Asian, *Plasmodium falciparum*, *Plasmodium vivax*

## Abstract

**Background:**

Glucose 6-phosphate dehydrogenase (G6PD) and pyruvate kinase (PKLR) deficiencies are common causes of erythrocyte haemolysis in the presence of antimalarial drugs such as primaquine and tafenoquine. The present study aimed to elucidate such an association by thoroughly investigating the haematological indices in malaria patients with *G6PD* and *PKLR*^*R41Q*^ variants.

**Methods:**

Blood samples from 255 malaria patients from Thailand, Myanmar, Laos, and Cambodia were collected to determine haematological profile, G6PD enzyme activity and *G6PD* deficiency variants. The multivariate analysis was performed to investigate the association between anaemia and *G6PD Mahidol*^*G487A*^, the most common mutation in this study.

**Results:**

The prevalence of G6PD deficiency was 11.1% (27/244) in males and 9.1% (1/11) in female. The MAFs of the *G6PD Mahidol*^*G487A*^ and *PKLR*^*R41Q*^ variants were 7.1% and 2.6%, respectively. Compared with patients with *wildtype G6PD* after controlling for haemoglobinopathies, G6PD-deficient patients with hemizygous and homozygous *G6PD Mahidol*^*G487A*^ exhibited anaemia with low levels of haemoglobin (11.16 ± 2.65 g/dl, *p* = 0.041). These patients also exhibited high levels of reticulocytes (3.60%). The median value of G6PD activity before treatment (Day 0) was significantly lower than that of after treatment (Day 28) (5.51 ± 2.54 U/g Hb vs. 6.68 ± 2.45 U/g Hb; *p* < 0.001). Reticulocyte levels on Day 28 were significantly increased compared to that of on Day 0 (2.14 ± 0.92% vs 1.57 ± 1.06%; *p* < 0.001). *PKLR*^*R41Q*^ had no correlation with anaemia in malaria patients. The risk of anaemia inpatients with *G6PD* *Mahidol*^*G487A*^ was higher than *wildtype* patients (OR = 3.48, CI% 1.24–9.75, *p* = 0.018). Univariate and multivariate analyses confirmed that *G6PD* *Mahidol*^*G487A*^ independently associated with anaemia (< 11 g/dl) after adjusted by age, gender, *Plasmodium* species, parasite density, *PKLR*^*R41Q*^, and haemoglobinopathies (*p* < 0.001).

**Conclusions:**

This study revealed that malaria patients with *G6PD Mahidol*^*G487A*^, but not with *PKLR*^*R41Q*^, had anaemia during infection. As a compensatory response to haemolytic anaemia after malaria infection, these patients generated more reticulocytes. The findings emphasize the effect of host genetic background on haemolytic anaemia and the importance of screening patients for erythrocyte enzymopathies and related mutations prior to anti-malarial therapy.

**Supplementary Information:**

The online version contains supplementary material available at 10.1186/s12936-022-04267-7.

## Background

Glucose 6-phosphate dehydrogenase (G6PD; EC 1.1.1.49) and pyruvate kinase (PKLR; EC:2.7.1.40) deficiencies are the most common hereditary metabolic disorders affecting red blood cells [1, 2]. G6PD deficiency triggers haemolytic anaemia in states of oxidative stress because deficient erythrocytes contain low levels of NADPH, which is required for maintaining cellular redox homeostasis through glutathione recycling [2]. Millions of people worldwide, mostly in Africa, the Mediterranean, the Middle East, and Asia, are affected by this condition. G6PD deficiency is caused by mutations in the *G6PD* gene on chromosome X. Genetically, males are either G6PD deficient or G6PD normal, while females can be homozygous with G6PD deficiency (mutations are present on both X chromosomes) or heterozygous (one X chromosome is affected) or G6PD normal. The frequency of *G6PD* status follows the Hardy Weinberg equilibrium. This makes heterozygous females are more common than hemizygous males and homozygous females are the least common [2].

Approximately 186 *G6PD* mutations, most of which are point mutations, have been documented [3]. Each mutation has different clinical phenotypes and distinctive geographical and ethnic distributions [4]. Recently, *G6PD Mahidol*^*G487A*^, a common Southeast Asian mutation, has been reported to reduce *Plasmodium vivax* density [5]. Additionally, a study in Afghanistan has demonstrates that G6PD deficiency protects against *P. vivax* clinical disease [6]. Even though G6PD deficiency provides clinical protection against *Plasmodium* spp., G6PD-deficient patients are susceptible to haemolytic anaemia when exposed to active and toxic metabolites of primaquine (PQ) and tafenoquine (TQ) [7–9]. PQ and TQ are anti-malarial drugs that reduce *Plasmodium falciparum* gametocytes for transmission and preventing the relapse of *P. vivax* and *Plasmodium ovale* malaria [8, 9]. In 2013, Howes et al. published the spatial distribution of G6PD deficiency and its mutations in malaria-endemic areas around the globe to support the safe use of PQ and TQ [10]. The diagnosis of G6PD deficiency and molecular genotyping of *G6PD* in malaria patients prior to PQ and TQ administration are necessary to prevent adverse outcomes [8].

PK deficiency (PKD), the second most common enzyme deficiency, causes haemolytic anaemia worldwide with an estimated prevalence of 0.005% (1/20,000) in the Caucasian population. The prevalence of PKD in the European population was estimated to be less than 0.05% (5/10,000) [1], 3.4% in the Hong Kong population and 2.2% in Chinese infants [11, 12]. The prevalence of PKD in Southeast Asian countries has yet to be determined. PKD is caused by loss-of-function mutations in *PK* predominantly expressed in the liver and red blood cells (*PKLR*). More than 150 mutations of *PKLR* have been reported [13]. Evidence in murine models has suggested that PKD confers a protective effect against malaria [14]. Recently, a novel point mutation (161A > G) resulting in an amino acid change at residue 41 from arginine (R), which is highly conserved in the PK family, to glutamine (Q) (R41Q) in the N-terminus of PK has been reported [15]. However, the haematological parameters in malaria patients with *G6PD* and *PKLR*^*R41Q*^ mutations have not been thoroughly investigated. The main aim of the present study was to examine the haematological profiles in malaria patients with *G6PD* or *PKLR* mutations.

## Methods

### Study subjects and sample collection

The study protocol was approved by the Institutional Review Board of the Faculty of Medicine, Chulalongkorn University (Bangkok, Thailand) (COA No. 040/2013, IRB No. 459/55). All patients were screened by passive case detection (PCD) protocol and provided written informed consent prior to enrollment in this study. A total of 255 uncomplicated malaria patients who were admitted to the Hospital for Tropical Diseases in Thailand during 2011–2012 with blood slide positivity for *Plasmodium* spp*.* and had no history of anti-malarial drug treatment 2 weeks prior were recruited for this study. Blood samples were collected at the Hospital for Tropical Diseases in Bangkok, Thailand and transferred on ice to a research laboratory at the Faculty of Medicine, Chulalongkorn University, Bangkok, Thailand within an hour for immediate measurement of G6PD activity and haematological parameters. Complete blood count (CBC) was measured using an BC-6800 Auto Hematology Analyzer (Mindray Medical International, China).

### Identify *Plasmodium* spp.

Giemsa staining of thick and thin blood smears prepared from finger pricks was examined every 12 h from initiation of treatment until they were negative. Blood smears were examined daily until patients were discharged. *Plasmodium* spp. were identified under a microscope by an independent parasitologist at the Hospital for Tropical Diseases and further confirmed by polymerase chain reaction (PCR)-based analysis [16].

### Measurement of G6PD activity

G6PD activity in the blood of malaria patients was measured in triplicate along with the normal and G6PD-deficient controls (G6888, G5888; Trinity Biotech, Ireland) using a quantitative assay kit for G6PD (Trinity Biotech, Ireland) prior to treatment and repeated weekly until patients were discharged. This assay measured NADPH production by G6PD in the blood of patients in parallel with positive and negative controls. Detection was carried out at a wavelength of 340 nm. The haemoglobin level was measured using Hb201 (HemoCue, Sweden) and used to calculate G6PD activity. Leftover blood samples were kept at − 20 °C for molecular typing.

### Identification of *G6PD*, *PKLR*^R41Q^, and thalassaemia mutations

Genomic DNA from frozen blood samples was extracted using the phenol–chloroform method. PCR–restriction fragment length polymorphism (PCR–RFLP) was used to identify *G6PD Mahidol*^*G487A*^*, G6PD Viangchan*^*G871A*^*,* and *G6PD Kaiping*^*G1388A*^*,* as described previously [17], *and PKLR*^*R41Q*^ according to a previous report [15]. *PKLR*^*R41Q*^ was amplified by PKLR-R41Q-F: 5'–GCC AAC GGG GTA TCT ACG GC–3' and PKLR-R41Q-R: 5'–GCA GAG GTG TTC CAG GAA GG–3'. After digestion with *Aci*I, the PCR product size of *PKLR*^*R41Q*^ was 121 base pairs (bp). The normal allele produced two fragments of 102 bp and 19 bp. For G6PD-deficient patients with unknown mutations by the PCR–RFLP method, the coding exons of *G6PD* (exons 3–12) were amplified using primers described previously [16]. The PCR products were sequenced (Macrogen, Korea). The sequencing data were analysed using BioEdit software version 2.1 with the *G6PD* reference sequence (GenBank accession number X55448.1).

For thalassaemia mutations, multiplex gap-polymerase chain reaction was performed as previously described with minor modifications [18] to detect *-α*^*3.7*^*, -α*^*4.2*^*, –*^*SEA*^ and *–*^*FIL*^ variants of the α-globin gene. PCR–RFLP was used to identify HbCS and HbE, as described previously [19, 20].

### Statistical analysis

All statistical analyses were performed using SPSS version 22 (IBM SPSS software, IL, USA). Data are expressed as percentages, median ± interquartile range (IQR), and mean ± standard deviation (SD). Following a computation approach reported previously, the adjusted male median (AMM) of G6PD activity was used to determine the cut-off values for G6PD deficiency [7]. The AMM values were defined as 100% activity of all male subjects after removing subjects with severe G6PD activity (≤ 10% of the overall median G6PD activity). The cut-off points for G6PD deficiency, G6PD intermediate (mild deficiency), and normal were median values less than 30%, 30 to 70%, and over 70% of the AMM, respectively. The G6PD activity of patients before (Day 0) and after treatment (Day 28) were compared using the Wilcoxon signed-rank test. The two-tailed Student’s *t*-test was used to analyse the differences in quantitative variables. Haemoglobin less than 11 g/dl is considered anaemia [21]. The risk of anaemia in G6PD status and mutations was analysed by odd ratio (OR). In a study of the association between haematological parameters, G6PD deficiency/G6PD normal and *G6PD Mahidol*^*G487A*^*/G6PD wildtype*, a multiple linear regression was performed adjusted for age, gender, parasite species, parasite density, and haemoglobinopathies. A statistically significant difference was defined as two-sided with a *p*-value less than 0.05.

## Results

### Demographic data and prevalence of *G6PD* and *PKLR*^R41Q^ mutations in malaria patients

**Fig. 1:**
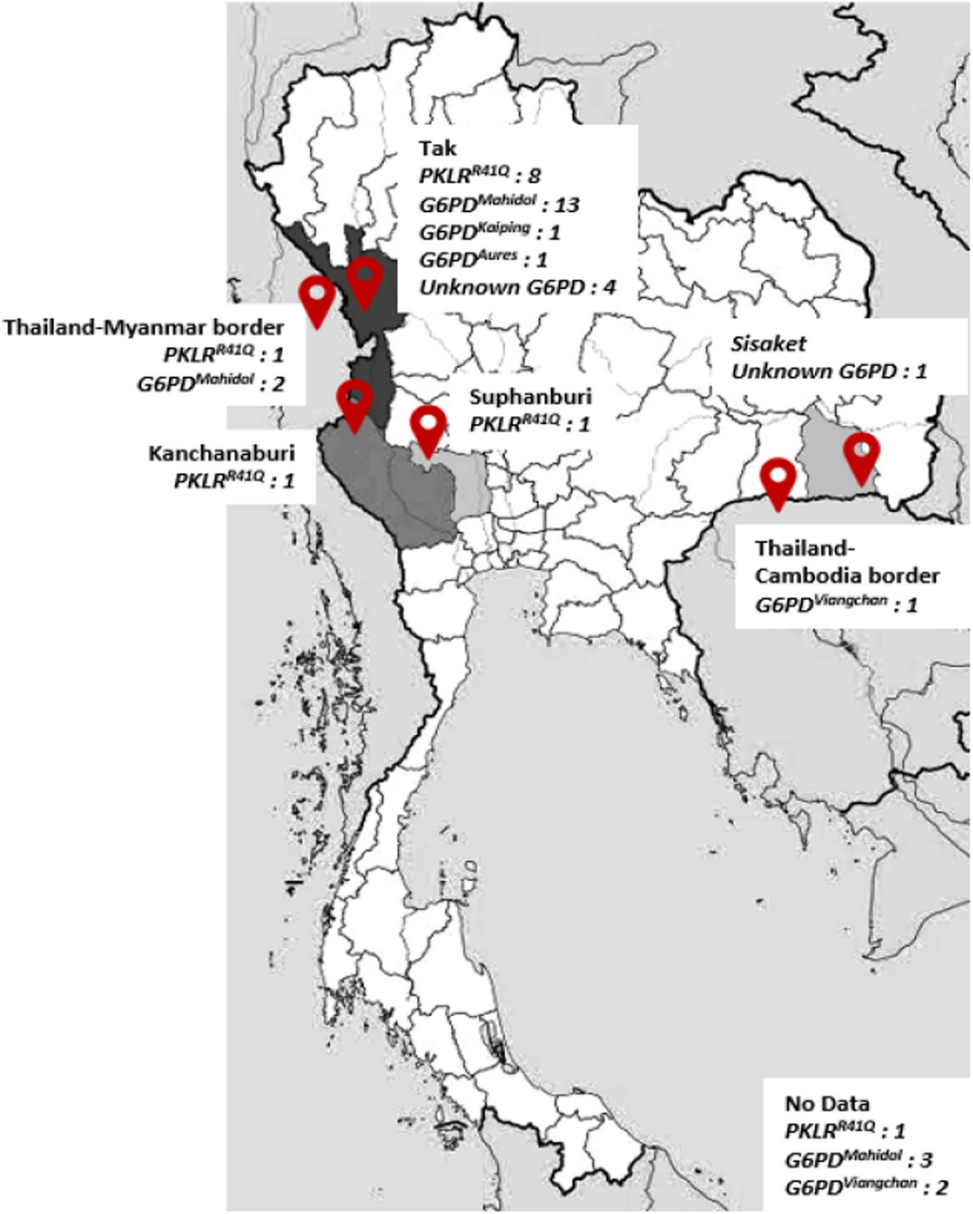
Geographic location of dominant variants found in a malaria patient cohort along Thailand and borders during 2011–2012

A total of malaria patients consisting of 189 individuals from Myanmar (74.1%), 35 Thais (13.7%), 16 Karen (6.3%), 8 Cambodians (3.1%), 4 Mons (1.6%), 2 Laotians (0.8%), and 1 unknown ethnicity (0.4%) are summarized in Table 1. These patients were from malaria-endemic areas, including the Thailand-Myanmar border (N = 25), Thailand-Cambodia border (N = 5) and several provinces of Thailand (N = 225), as described in a previous report [16] (Fig. 1). Two hundred forty-four patients (95.7%) were male, and eleven patients (4.3%) were female. The skewness of the gender ratio was influenced by male labor migration. The mean age of all patients was 27.94 ± 9.93 years (range 14–60 years). The numbers of patients infected with *P. falciparum* and *P. vivax* were 106 (41.6%) and 145 (56.9%), respectively. Three patients (1.2%) had coinfection of *P. falciparum* and *P. vivax*. One patient (0.4%) was infected with *Plasmodium malariae*.

**Table 1:** Summary of study population characteristics

	Number of Enrolled	Myanmarese (%)	Cambodian (%)	Karen (%)	Laotians (%)	Mons (%)	Thais (%)	Unknown (%)	Total (%)
Ethnicity	Malaria patients	189 (74.1)	8 (3.1)	16 (6.3)	2 (0.8)	4 (1.6)	35 (13.7)	1 (0.4)	255
Age	Median ± IQR (years)	25.0 ± 15.0	24.5 ± 6.0	20.0 ± 7.0	28.0	21.5 ± 6.0	29.5 ± 15.0	16.0	25.0 ± 14.0
	Range (years)	14–057	19–30	14–40	24–32	19–26	16–60	16	14–60
Sex	Male (%)	182 (96.3)	8 (100)	16 (100)	2 (100.0)	4 (100.0)	31 (88.6)	1 (100.0)	244 (95.7)
	Female (%)	7 (3.7)	–	–	–	–	4 (11.4)	–	11 (4.3)
*Plasmodium spp.*	*P. falciparum* (%)	81 (42.9)	1 (12.5)	7 (43.8)	2 (100.0)	1 (25.0)	14 (40.0)	–	106 (41.6)
	*P. vivax* (%)	105 (55.6)	6 (75.0)	9 (56.3)	–	3 (75.0)	21 (60.0)	1(100.0)	145 (56.9)
	*Co-infection (Pf* + *Pv)* (%)	2 (1.1)	1 (12.5)	–	–	–	–	–	3 (1.2)
	*P. malariae* (%)	1 (0.5)	–	–	–	–	–	–	1 (0.4)
*G6PD* (n = 45)	G6PD deficiency (%)	19 (10.1) M:18, F:1	1 (12.5) M:1	3 (18.8) M:3	–	–	5 (14.3) M: 5	–	28 (11.0) M: 27 (11.1), F: 1 (9.1)
	G6PD intermediate (%)	11 (5.8) M: 10, F: 1	–	1 (6.3) M: 1	–	–	5 (14.3) M: 3, F: 2	**–**	17 (6.7) M: 14 (5.7), F: 3 (27.3)
	*Mahidol*^*G487A*^ mutation (%)	14 (7.4) (Hemi: 13) (Homo: 1)	–	2 (12.5) (Hemi: 2)	–	–	2 (5.7) (Hemi: 2)	**–**	18 (7.1) (Hemi: 17, Homo: 1)
	*MAF*								
	Overall								
	G allele (%)	181 (92.3)	8 (100.0)	14 (87.5)	2 (100.0)	4 (100.0)	37 (94.9)	1 (100.0)	242 (92.9)
	A allele (%)	15 (7.7)	–	2 (12.5)	–	–	2 (5.1)	–	19 (7.1)
	Male only								
	G allele (%)	169 (92.9)	8 (100.0)	14 (87.5)	2 (100.0)	4 (100.0)	29 (93.6)	1 (100.0)	227 (93.0)
	A allele (%)	13 (7.1)	–	2 (12.5)	–	–	2 (6.4)	–	17 (7.0)
	*Viangchan*^*G871A*^ mutation (%)	–	1 (100.0) (Hemi:1)	–	–	–	2 (5.7) (Hemi: 2)	–	3 (1.2) (Hemi: 3)
	*Kaiping*^*G1388A*^ mutation (%)	–	–	1 (6.3) (Hemi: 1)	–	–	–	–	1 (0.4) (Hemi: 1)
	*Aures*^*T143C*^ mutation (%)	1 (0.5) (Hemi: 1)	–	–	–	–		–	1 (0.4) (Hemi: 1)
	Unknown mutation (%)	4 (2.1)	–	–	–	–	1 (2.9)	–	5 (2.0)
*PKLR* (n = 12)	*PKLR*^*R41Q*^ mutation (%)	9 (4.8)	1 (12.5)	–	–	–	2 (5.7)	–	12 (4.7)
	Genotype G A (%)	8 (88.9)	1 (100.0)	–	–	–	2 (100.0)	–	11 (4.3)
	Genotype A A (%)	1 (11.1)	–	–	–	–	–	–	1 (0.4)
	*MAF*								
	G allele (%)	368 (97.4)	15 (93.7)	32 (100.0)	4 (100.0)	8 (100.0)	68 (97.1)	2 (100.0)	497 (97.5)
	A allele (%)	10 (2.6)	1 (6.3)	–	–	–	2 (2.9)	–	13 (2.6)

**Fig. 2:**
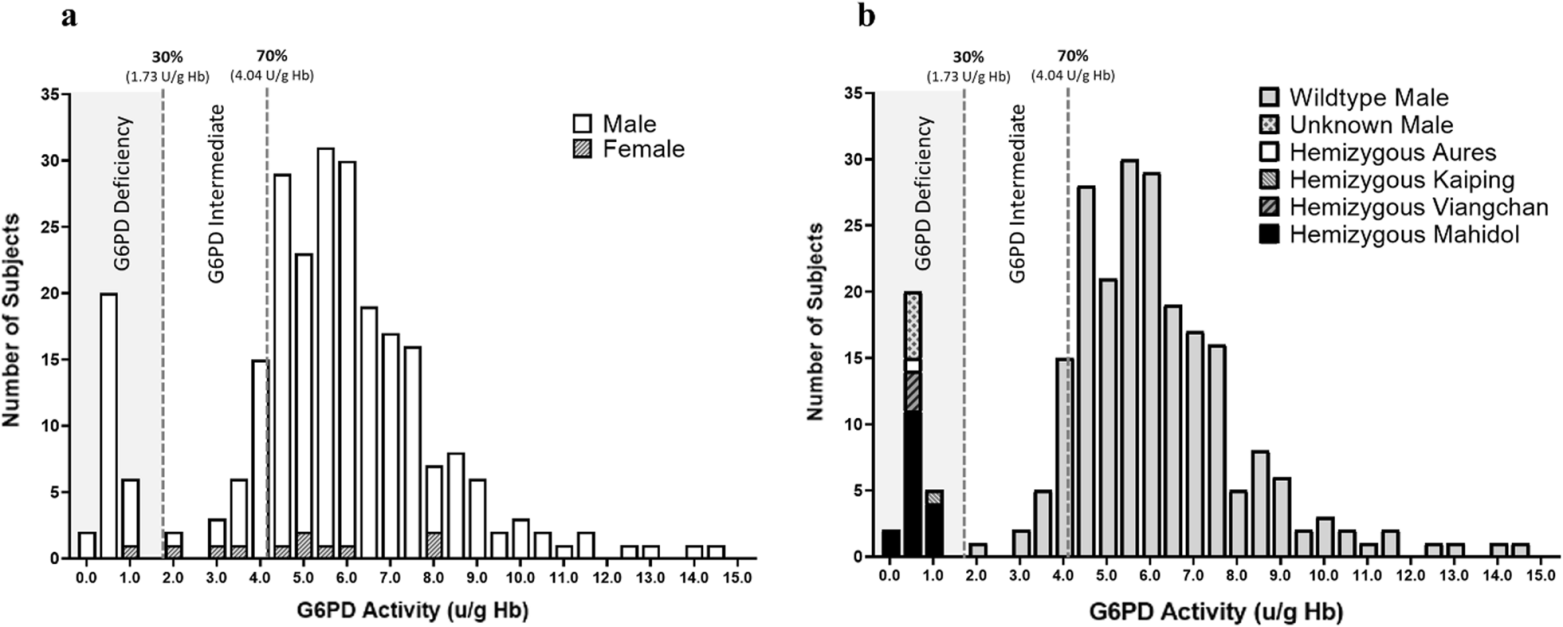
The histograms show the distribution of G6PD activity (U/g Hb); **a** G6PD activity (U/g Hb) in the study population (male and female) and **b** G6PD activity (U/g Hb) in individual G6PD genotypes in males. G6PD activity less than 1.73 U/g Hb (< 30% AMM), between 1.73 and 4.04 U/g Hb (30–70% AMM), and more than 4.04 U/g Hb (> 70% AMM) were indicated G6PD deficiency, G6PD intermediate, and G6PD normal, respectively

The overall median value of G6PD activity in this cohort (n = 255) was 5.66 ± 2.55 U/g Hb (median ± IQR), ranging from 0.00 to 14.59 U/g Hb. The median values of G6PD activity in males (n = 244) and in females (n = 11) were 5.68 ± 2.49 and 4.80 ± 3.10 U/g Hb, ranging from 0.00 to 14.59 U/g Hb and 1.04 to 7.95 U/g Hb, respectively. The adjusted male median (AMM) G6PD activity in G6PD normal was 5.77 U/g Hb. The cut-off values for G6PD deficiency and G6PD intermediate were < 1.73 U/g Hb (< 30% of the AMM) and 4.04 U/g Hb (30–70% of the AMM), respectively (Fig. 2). G6PD activity exhibited bimodal distribution in males and normal distribution in females. The median values of G6PD activity in G6PD deficiency and G6PD intermediate were 0.49 ± 0.39 U/g Hb (range from 0.00 to 1.04 U/g Hb) and 3.59 ± 0.82 U/g Hb (range from 1.86 to 4.04 U/g Hb) (Fig. 2). According to these cut-off values, 27 male (11.1%; of a total of 244) and 1 female (9.1%; of a total of 11) patients were identified as G6PD deficient. The prevalence of G6PD intermediate in this study was 5.7% (14/ 244) in males and 27.3% (3/ 11) in females. Of 45 patients with G6PD deficiency and intermediate G6PD, 19 patients (42.2%) were infected with *P. falciparum*, 25 patients (55.6%) were infected with *P. vivax*, and 1 patient (2.2%) was coinfected with *P. falciparum* and *P. vivax*. Of all 28 patients with G6PD deficiency, 18 patients carried *G6PD Mahidol*^*G487A*^ (17 hemizygous deficient males, 1 homozygous deficient female), 3 patients carried *G6PD Viangchan*^*G871A*^ (3 hemizygous deficient males), 1 patient carried *G6PD Kaiping*^*G1388A*^ (1 hemizygous deficient male) and 1 patient carried *G6PD Aures*^*T143C*^ (1 hemizygous deficient male) (Table 1). In the remaining 5 patients, both PCR–RFLP and DNA sequencing methods could not detect mutations in the *G6PD* gene. The minor allele frequency (MAF) of *G6PD Mahidol*^*G487A*^ was 7.1% in these populations (Table 1). Individually, 17 hemizygous *G6PD Mahidol*^*G487A*^ and 3 hemizygous *G6PD Viangchan*^*G871A*^ were 0.40 ± 0.46 U/g Hb (range from 0.00 to 0.89 U/g Hb) and 0.63 U/g Hb (range from 0.31 to 0.66 U/g Hb), respectively. G6PD activity levels in 1 hemizygous male with *G6PD Kaiping*^*G1388A*^, 1 hemizygous male with *G6PD Aures*^*T143C*^, and 1 homozygous female with *G6PD Mahidol*^*G487A*^ were 1.02 U/g Hb, 0.40 U/g Hb, and 1.04 U/g Hb, respectively.

The *PKLR*^*R41Q*^ mutation was detected in 12 patients (4.7%). These patients were from Thailand and Thailand borders (Fig. 1). The number of patients with *PKLR*^*R41Q*^ who were infected with *P. falciparum* and *P. vivax* was equal. As shown in Table 1, *PKLR*^R41Q^ was detected in malaria patients with an MAF of 2.6% in the study population (13 of 510), 2.6% in individuals from Myanmar (10 of 378), 2.9% in Thais (2 of 70) and 6.3% in Cambodians (1 of 16).

### Haematological profiles of malaria patients with *G6PD *and *PKLR *mutations

At the first visit prior malaria treatment, malaria patients with G6PD deficiency (n = 28), compared to malaria patients with normal G6PD activity levels (n = 209), exhibited a significant decrease in the haemoglobin levels (11.03 ± 2.51 g/dl vs. 12.65 ± 1.97 g/dl; *p* = 0.003). These patients also had a significant increase in the reticulocyte count (2.80 ± 2.05% vs. 1.49 ± 1.07%; *p* = 0.005). Malaria patients with *G6PD Mahidol*^*G487A*^ mutation (n = 17) compared to wildtype patients without common Southeast Asian (SEA) mutations including the *G6PD Mahidol*^*G487A*^ (n = 215) exhibited a significant decrease in haemoglobin levels (11.16 ± 2.65 g/dl vs. 12.66 ± 1.92 g/dl; *p* = 0.041). These patients also had an increase of reticulocyte levels (3.61 ± 2.44% vs. 1.47 ± 1.05%; *p* = 0.008) (Table 2). There were not statistically differences in malaria patients with *PKLR*^*R41Q*^ compared to those with wildtype. The coexistence of G6PD deficiency and thalassaemia/haemoglobinopathies is very common in this region. One hundred twenty-nine malaria patients with thalassaemia and haemoglobinopathies were found in this study population. After excluding these patients, an association between G6PD deficiency and anaemia in malaria patients was found. The data were shown in Additional file 1: Table S1.

**Table 2:** Mean and standard deviation (SD) for clinical parameters of malaria patients with erythrocytic enzymopathies (*p*-values were determined using the Student’s *t*-test.)

Clinical parameters	G6PD Phenotypic status (n = 255, missing 1)					*G6PD* and *PKLR* mutations (n = 255, missing 1)							
(n = 255)	G6PD normal	G6PD intermediate	*p*	G6PD deficiency	*p*	Wild-type	Mahidol	*p*	Other *G6PD* mutations	*p*	*PKLR*^R41Q^	*p*	*Mahidol* + *PKLR*^R41Q^
	(n = 209)	(n = 17)		(n = 28)		(n = 215)	(n = 17)		(n = 10)		(n = 11)		(n = 1)
Hb	12.7 ± 2.0	13.2 ± 1.1	0.084	11.0 ± 2.5	**0.003**	12.7 ± 1.9	11.2 ± 2.7	**0.041**	10.6 ± 2.4	**0.001**	13.2 ± 1.8	0.353	13.1
(g/dl)	(6.4–17.0)	(11.6–15.3)		(6.5–15.3)		(6.4–17.0)	(6.5–15.2)		(7.8–15.3)		(8.2–15.3)		
RBCs	4.9 ± 0.8	5.0 ± 0.5	0.542	4.3 ± 1.0	**0.005**	4.8 ± 0.8	4.0 ± 0.9	**<0.001**	4.6 ± 1.0	0.417	5.2 ± 0.5	0.100	4.95
(× 10^6^/ul)	(1.3–7.1)	(4.3–5.8)		(2.2–6.0)		(1.3–7.1)	(2.2–5.3)		(2.7–6.0)		(4.5–6.3)		
Hct	38.4 ± 5.7	40.2 ± 4.9	0.060	34.17 ± 7.1	**0.006**	38.4 ± 5.5	34.0 ± 7.2	**0.029**	33.9 ± 7.4	**0.014**	41.2 ± 5.3	0.094	39.9
(%)	(19.4–52.8)	(35.3–48.4)		(20.7–44.7)		(19.4–52.8)	(20.7–44.7)		(22.0–44.4)		(29.7–47.4)		
MCV	79.3 ± 8.6	81.4 ± 6.7	0.327	81.2 ± 8.9	0.281	79.5 ± 8.4	85.8 ± 7.3	**0.004**	74.1 ± 6.9	**0.046**	79.3 ± 9.7	0.938	80.6
(fl)	(47.8–96.5)	(66.0–88.8)		(62.5–95.9)		(47.8–96.5)	(71.4–95.9)		(62.5–82.2)		(58.9–90.0)		
MCH	26.3 ± 3.3	26.7 ± 1.7	0.404	26.2 ± 3.7	0.879	26.3 ± 3.2	28.0 ± 2.8	**0.046**	23.2 ± 3.3	**0.002**	25.5 ± 3.68	0.371	26.6
(pg/cell)	(16.2–38.5)	(23.2–29.3)		(17.3–30.7)		(16.3–38.5)	(23.1–30.7)		(17.3–29.1)		(16.2–29.3)		
MCHC	32.8 ± 2.4	32.8 ± 1.4	0.975	31.0 ± 6.0	0.137	32.9 ± 2.4	30.7 ± 7.6	0.269	31.4 ± 2.5	0.055	32.0 ± 2.8	0.239	33.0
(g/dl)	(9.0–36.6)	(30.9–35.8)		(3.0–36.6)		(9.00–36.6)	(3.0–36.6)		(26.9–35.4)		(27.5–35.0)		
RDW	15.2 ± 1.6	14.7 ± 0.7	**0.015**	14.9 ± 2.2	0.387	15.1 ± 1.5	14.4 ± 1.4	0.054	15.7 ± 3.1	0.558	15.8 ± 2.2	0.319	15.3
(%)	(12.7–23.8)	(13.4–16.2)		(12.3–23.8)		(12.7–23.8)	(12.3–17.5)		(12.8–23.8)		(13.1–19.1)		
Reticulocyte	1.5 ± 1.1	1.1 ± 0.4	0.120	2.8 ± 2.1	**0.005**	1.5 ± 1.1	3.6 ± 2.4	**0.008**	1.9 ± 0.9	0.225	1.5 ± 1.0	0.812	1.5
(%)	(0.3–7.8)	(0.5–2.0)		(0.5–9.6)		(0.3–7.8)	(1.6–9.6)		(0.5–3.0)		(0.7–3.8)		
Platelet	107.2 ± 82.3	73.71 ± 56.1	0.102	111.26 ± 57.8	0.803	105.6 ± 83.0	131.8 ± 60.0	0.215	83.0 ± 41.2	0.392	84.0 ± 31.2	0.390	65
(× 10^3^/mm^3^)	(14.0–790.0)	(7.0–217.0)		(11.0–275.0)		(7.0–790.0)	(52.0–275.0)		(11.0–150.0)		(44.0–146.0)		
MPV	13.3 ± 52.1	9.6 ± 3.1	0.770	9.3 ± 1.4	0.693	13.2 ± 51.3	9.5 ± 1.5	0.773	9.0 ± 1.9	0.795	8.4 ± 0.9	0.754	9.1
(fl)	(0.0–738.0)	(0.0–13.6)		(7.2–13.5)		(0.0–738.0)	(7.2–13.5)		(7.4–10.9)		(7.5–10.5)		
TB	1.3 ± 2.0	3.3 ± 9.0	0.357	1.4 ± 0.8	0.668	1.4 ± 3.2	1.6 ± 0.9	0.875	1.3 ± 0.7	0.862	0.9 ± 0.5	0.548	0.55
(mg/dl)	(0.2–21.3)	(0.4–38.0)		(0.3–3.3)		(0.2–37.98)	(0.3–3.3)		(0.5–2.4)		(0.3–2.0)		
DB	0.6 ± 1.6	2.3 ± 7.9	0.377	0.6 ± 0.5	0.915	0.8 ± 2.8	0.6 ± 0.6	0.863	0.5 ± 0.3	0.753	0.4 ± 0.2	0.591	0.29
(mg/dl)	(0.0–17.7)	(0.2–33.0)		(0.0–2.4)		(0.0–33.0)	(0.1–2.4)		(0.04–1.06)		(0.1–0.7)		
IDB	0.6 ± 0.5	0.9 ± 1.3	0.305	0.8 ± 0.5	0.055	0.6 ± 0.6	0.9 ± 0.6	0.120	0.7 ± 0.4	0.617	0.5 ± 0.3	0.461	0.26
(mg/dl)	(0.1–3.6)	(0.2–5.0)		(0.1–2.5)		(0.0–5.0)	(0.1–2.5)		(0.3–1.67)		(0.2–1.3)		
Parasitemia	27,419.2 ± 38,747.8	33,379.0 ± 59,709.0	0.691	24,841.9 ± 26,411.5	0.738	27,988.3 ± 41,225.2	25,633.9 ± 24,868.7	0.822	25,982.3 ± 30,289.8	0.879	24,681.4 ± 25,455.0	0.793	765
(parasite/ul)	(35–278,250)	(1–185,820)		(133–81,900)		(1–278,250)	(133–72,750)		(800–81,900)		(1018–69,940)		

Hb: haemoglobin; RBCs: red blood cells; Hct: haematocrit; MCV: mean corpuscular volume; MCH: mean corpuscular haemoglobin; MCHC: mean corpuscular Hb concentration; WBCs: white blood cells; RDW: red cell distribution width; TB: total bilirubin; DB: direct bilirubin; IDB: indirect bilirubin; Bold values indicate statistical significance at the *p*-value <0.05 level

**Fig. 3:**
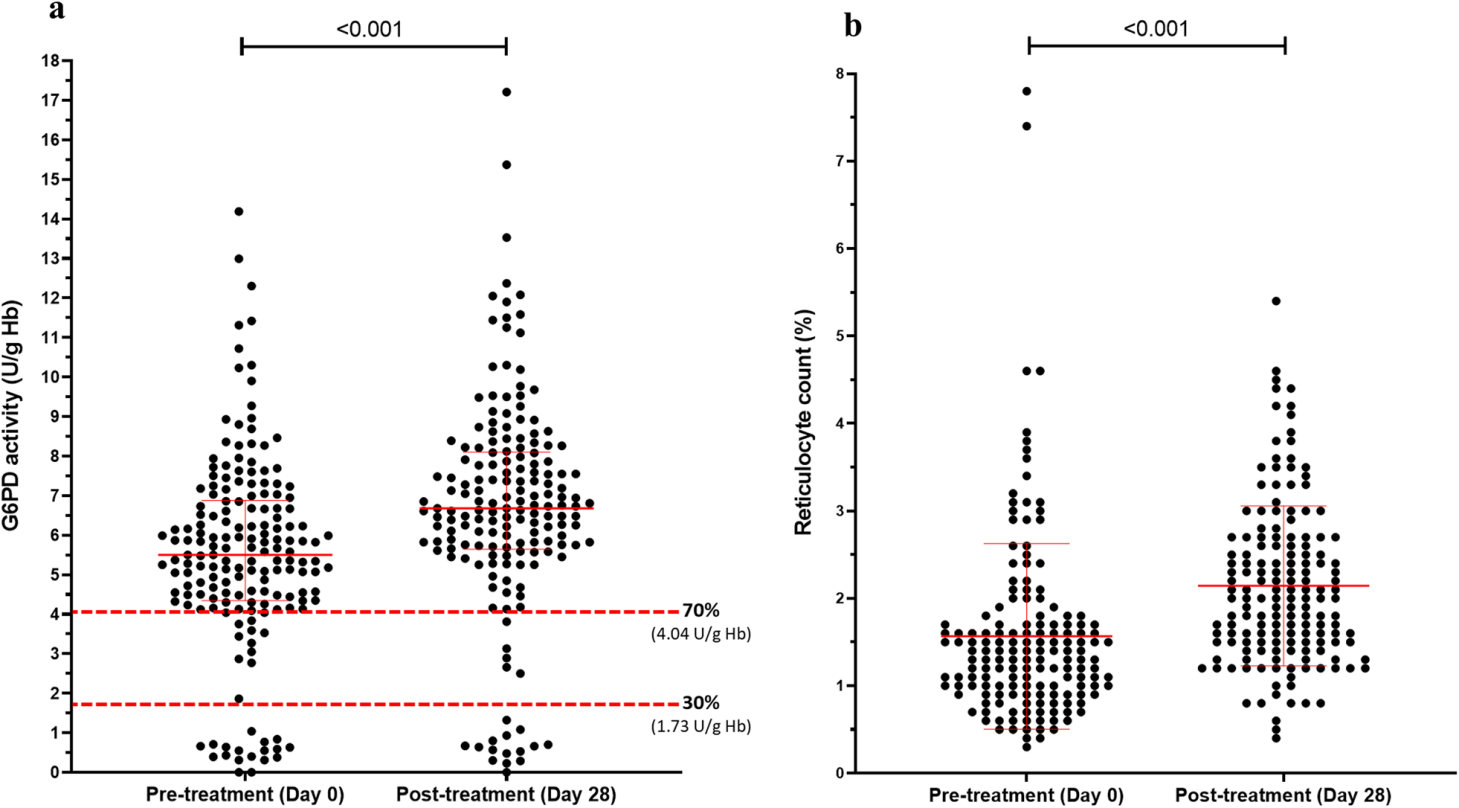
Scatter plots of paired **a** G6PD activity (median ± IQR) and **b** reticulocyte levels (mean ± SD) on Day 0 and Day 28 of treatment

In the longitudinal monitoring of G6PD activity before (Day 0) and after (Day 28) treatment, there was a total of 174 malaria patients with complete haematological data were analysed. These patients included G6PD deficiency, thalassaemia, and haemoglobinopathies. The median value of G6PD activity on Day 28 was significantly higher than that of on Day 0 (6.68 ± 2.45 U/g Hb vs. 5.51 ± 2.54 U/g Hb; *p* < 0.001) (Fig. 3a). In G6PD normal and intermediate group, the median values of G6PD activity on Day 28 were significantly increased, compared with that of on Day 0 (G6PD normal: 7.34 ± 2.39 U/g Hb vs. 6.32 ± 2.15 U/g Hb; *p* < 0.001; G6PD intermediate: 6.62 ± 1.59 U/g Hb vs. 3.49 ± 0.90 U/g Hb; *p* < 0.001). The median value of G6PD activity in G6PD deficiency group on Day 28 was significantly different from that of on Day 0 (0.67 ± 0.71 U/g Hb vs. 0.55 ± 0.31 U/g Hb; *p* = 0.043). Additionally, reticulocyte levels on Day 28 were significantly increased compared to that of on Day 0 (2.14 ± 0.92% vs 1.57 ± 1.06%; *p* < 0.001) (mean ± SD) (Fig. 3b). In groups of G6PD normal and G6PD intermediate, the mean values were significantly increased on Day 28 compared with that of on Day 0 (G6PD normal: 2.15 ± 0.95% vs. 1.51 ± 1.10%; *p* < 0.001; G6PD intermediate: 2.06 ± 0.55% vs. 1.24 ± 0.41%; *p* = 0.001). However, there were not statistically significant in reticulocyte count between Day 0 and Day 28 in G6PD deficiency group.

Anaemia according to the most commonly used in malaria studies can be grouped into three categories base on haemoglobin concentrations including mild anaemia (Hb < 11 g/dl), moderate anaemia (Hb < 8 g/dl), and severe anaemia (Hb < 5 g/dl) [21]. Mild to moderate anaemia was found in 50.0% (14 of 28 cases) of G6PD-deficient individuals and 17.7% (37 of 209 cases) of normal patients (OR = 4.65; CI%: 2.04–10.57, *p* < 0.001). Besides, mild to moderate anaemia was observed in 41.2% (7 of 17 cases) of patients with *G6PD Mahidol*^*G487A*^ and 16.7% (36 of 215 cases) of patient with wildtype G6PD (non-common SEA mutation) (OR = 3.48; CI%: 1.24 – 9.75, *p* = 0.018). The univariate analysis revealed that haemoglobin levels were significantly associated with both G6PD deficiency/G6PD normal (*p* < 0.001) and *G6PD Mahidol*^*G487A*^*/ G6PD Wildtype* (*p* < 0.001) (Table 3). After adjusting by various variables including age, gender, *Plasmodium* species, parasite density, *PKLR*^*R41Q*^, thalassaemia and haemoglobinopathies, the multivariate analysis revealed that haemoglobin levels were significantly associated with G6PD deficiency/G6PD normal (*p* < 0.001) and *G6PD Mahidol*^*G487A*^*/ G6PD Wildtype* (*p* < 0.001) (Table 3).

**Table 3:** The univariate and multivariate analyses of G6PD deficiency/G6PD normal, *G6PD Mahidol*^*G487A*^*/G6PD wildtype* and haemoglobin levels adjusted by age, gender, parasite species, parasite density, *PKLR*^*R41Q*^, thalassaemia and haemoglobinopathies

Analysis type	Variable	Haemoglobin				Variable	Haemoglobin			
		B*	SE	Beta	*p*-value		B*	SE	Beta	*p*-value
Univariate										
	G6PD deficiency/G6PD normal	− 1.619	0.417	− 0.248	**< 0.001**	*G6PD* Mahidol/*G6PD* wildtype	− 1.415	0.495	− 0.182	**0.005**
	Gender	− 0.773	0.66	− 0.074	0.243	Gender	− 0.773	0.66	− 0.074	0.243
	Age	0.002	0.014	0.008	0.905	Age	0.002	0.014	0.008	0.905
	Parasite count	5.492 × 10^6^	0.000	0.106	0.095	Parasite count	5.462 × 10^6^	0.000	0.106	0.095
	Parasite species (*P. falciparum*/*P. vivax*)	− 0.485	0.264	− 0.117	0.067	Parasite species (*P. falciparum*/*P. vivax*)	− 0.485	0.264	− 0.117	0.067
	*PKLR*^*R14Q*^	0.723	0.605	0.076	0.233	*PKLR*^*R14Q*^	0.723	0.605	0.076	0.233
	Thalassaemia, haemoglobinopathies	− 0.467	0.258	− 0.114	0.072	Thalassaemia, haemoglobinopathies	− 0.467	0.258	− 0.114	0.072
Multivariate										
	G6PD deficiency/G6PD normal	− 1.895	0.447	− 0.270	**< 0.001**	*G6PD* Mahidol/*G6PD* wildtype	− 2.01	0.555	− 0.232	**< 0.001**
	Gender	− 1.292	0.780	− 0.108	0.099	Gender	− 0.865	0.643	− 0.088	0.18
	Age	0.002	0.014	0.011	0.871	Age	0.009	0.013	0.042	0.525
	Parasite count	1.011 × 10^5^	0.000	0.183	**0.008**	Parasite count	9.231 × 10^6^	0.000	0.179	**0.009**
	Parasite species (*P. falciparum*/*P. vivax*)	− 0.753	0.292	− 0.176	**0.011**	Parasite species (*P. falciparum*/*P. vivax*)	− 0.594	0.277	− 0.145	**0.033**
	*PKLR*^*R14Q*^	0.862	0.616	0.089	0.164	*PKLR*^*R14Q*^	0.766	0.574	0.085	0.184
	Thalassaemia, haemoglobinopathies	− 0.496	0.271	− 0.117	0.069	Thalassaemia, haemoglobinopathies	− 0.491	0.259	− 0.122	0.060
	Constant	14.567	1.196		**< 0.001**	Constant	13.883	1.064		**< 0.001**

Bold values indicate statistical significance at the *p* -value <0.05 level

## Discussion

Malaria infection causes haemolysis of infected erythrocytes. PQ and TQ, 8-aminoquinoline, are essential anti-malarial drugs commonly used for radical cure of *P. vivax* infections. It also reduces transmission of *P. falciparum*. However, PQ and TQ cause acute haemolytic complications in patients with G6PD deficiency. Although there have been many reports of G6PD deficiency status and its mutations in malaria patients living in the Southeast Asia, data on the haematological parameters of *G6PD* and other erythrocytic mutations including PK in malaria patients are limited. These data may be beneficial for the administration of anti-malarial treatment especially PQ and TQ prescription.

The overall frequency of patients with *P. vivax* infection was slightly higher than that of with *P. falciparum* infection, confirming that the frequencies of *P. vivax* and *P. falciparum*-infected cases are approximately equal, with high chances of coinfection in the international border between the territory of Myanmar and the western region of Thailand [22, 23]. Overall, 28 (11.0%) participants were G6PD deficient, which was presented as 11.1% (27/244) of males and 9.1% (1/11) of females. This finding is consistent with previous studies reporting that G6PD deficiency was found in approximately 10.0–13.7% of the male population of these ethnic groups [24, 25]. Although, the prevalence of G6PD deficiency in females (9.1%) in this population was higher than that reported previously (5.3%) [26], as a result of the small number of the female patients, the frequency of *G6PD* mutation in females follows Hardy–Weinberg equilibrium. Based on the population wide AMM, a total of 17 individuals (6.7%) exhibited intermediate G6PD activity, which was present in 5.7% (14/244) of males and 27.3% (3/11) of females. According to the results of this study and previous report, G6PD deficiency was more common in males than in females whereas intermediate was more common in females than in males [27].

These results showed that the *G6PD Mahidol*^*G487A*^ mutation was more common among individuals from Myanmar, in Thai, and in Karen malaria patients, whereas *G6PD Viangchan*^*G871A*^ mutation was more common among Thai and Cambodian malaria patients. In general, *G6PD* *Viangchan*^*G871A*^ is more common in Thai people than *G6PD Mahidol*^*G487A*^ [26]. Genetic admixture could explain the equal prevalence of *G6PD Viangchan*^*G871A*^ and *G6PD Mahidol*^*G487A*^ in this Thai population. The *G6PD Mahidol*^*G487A*^ mutation in malaria patients had an MAF of 7.1% in the study population (18 of 255), 7.4% in individuals from Myanmar (14 of 189), 5.7% in Thais (2 of 35) and 12.5% in Karen (2 of 16). These findings agreed with the spatial distribution of G6PD deficient mutations in the Southeast Asia, where *G6PD Mahidol*^*G487A*^ and *G6PD Viangchan*^*G871A*^ mutations are commonly observed on the western and eastern Indochina Peninsula, respectively [5, 17, 26, 28]. Although the *G6PD* genotype is a key factor of enzyme activity [29], some G6PD-deficient patients were unable to detect any mutations in *G6PD* coding regions. This could be explained by the methylation of CpG or CpNpG islands on *G6PD* promotor, resulting in gene silencing [30, 31]. Another possibility is that the presence of mutation in the 5’untranslated region (UTR) that has been reported to reduce enzyme activity [32].

The frequency of PK in this Southeast Asian population was comparable to what was reported by van Bruggen et al., who found this mutation in 13 out of 340 healthy unrelated Southeast Asian subjects with an MAF of 3.2% in individuals from Myanmar, 1% in Thais, 1.5% in Cambodians, 1.8% in Laotians, and 2.9% in Mons [15]. Possible contributing factors for the discrepancy between these findings include the differences in population size, homogeneity within each ethnic group and the place of origin of each subject (malaria vs non-malaria endemic areas).

Correlations between altered G6PD activity due to mutations in malaria patients and haematological phenotypes prior to treatment with anti-malarial drugs have not been well studied. Based on the International Classification of Diseases, 11th Revision (ICD-11) considering classification of G6PD deficiency under haemolytic anaemias (code: 3A10.00) [33], the data demonstrated that malaria patients with G6PD deficiency prior to treatment, particularly the *G6PD Mahidol*^*G487A*^ mutation, displayed signs of haemolytic anaemia, including low haemoglobin, RBC count, haematocrit, and high reticulocyte count. However, this study showed no signs of haemolytic anaemia in other *G6PD* and *PKLR*^*R41Q*^ mutations. This is possibly due to the small number of patients enrolled, which limit the chance to detect haemolytic anaemia in malaria patients carrying *G6PD Aures*^*T143C*^, *G6PD Viangchan*^*G871A*^ and *G6PD Kaiping*^*G1388A*^. According to the World Health Organization (WHO), G6PD variants are categorized based on the degree of enzyme deficiency and severity of haemolysis. *G6PD Mahidol*^*G487A*^ and *G6PD Aures*^*T143C*^ are in a class III mutation (moderately deficient) and *G6PD Viangchan*^*G871A*^ and *G6PD Kaiping*^*G1388A*^ are in a class II mutation (severely deficient) [3]. Increased G6PD activity levels after treatment in G6PD intermediate and normal groups was associated with reticulocytosis. The underlying mechanism for this phenomenon includes a post-treatment response of the bone marrow, which is suppressed during malaria infection [34–39].

*G6PD Mahidol*^*G487A*^ was an independent risk factor for anaemia based on age, gender, parasite species, parasite density, *PKLR*^*R41Q*^, thalassaemia, and haemoglobinopathies. G6PD-deficient RBCs are exposed to oxidative stress caused by active neutrophil-produced ROS [40], leading to a decline in haemoglobin levels and generates reticulocytes [41]. According to the *in-silico* study by Bharti et al., G6PD enzymes with the *Mahidol*^*487A*^ mutation lose their crucial catalytic interaction with substrate [42]. In addition, Boonyuen et al. have reported that *G6PD Mahidol*^*G487A*^ causes a local conformational change and affects backbone folding. This results in a reduction in thermostability in the absence or presence of NADP^+^ and a reduction in *K*_cat_, thereby reducing catalytic efficiency [42, 43]. The ability of erythrocytes to produce NADPH is diminished. NADPH is a reducing cofactor of glutathione reductase (GR), which reduces oxidized glutathione (GSSG) to reduced glutathione (GSH). GSH maintains the reduced state of the sulfhydryl group of haemoglobin and membrane proteins. In erythrocytes with the *G6PD Mahidol*^*487A*^ mutation, oxidation of membrane proteins causes the cells to rigid, nondeformable, and finally haemolysis. A recent report has indicated that patients with *G6PD Mahidol*^*G487A*^ presented symptoms of acute haemolytic anaemia after taking an incorrect dose of PQ [44]. Although this study had no haematological data to support the clinical impact of anti-malarial drugs on patients with *G6PD Mahidol*^*G487A*^ and other mutations, these findings provided evidence for malaria infection-induced haemolysis in patients with G6PD deficiency. This may be useful for G6PD deficiency testing requirements and administration of anti-malarial drugs including PQ and TQ to prevent relapse of *P. vivax* and sterilize mature *P. falciparum* gametocytes with a low risk of adverse events. For radical cure of *P. vivax* in the Southeast Asia, a high daily dose of PQ (0.5 mg/kg/day) for 14 days has been recommended by the WHO [45]. In Thailand and Cambodia, the lower 0.25 mg/kg daily for 14 days given as directly observed treatment (DOT) with G6PD deficiency testing has been recommended, whereas 0.5 mg/kg daily for 14 days at health centres and 0.75 mg/kg once a week for 8 weeks given as DOT since 2014 by malaria volunteers in the community has been recommended in Myanmar [46]. However, testing G6PD deficiency before PQ treatment was not routinely implemented in Myanmar and Cambodia but with poor implementation in Thailand [45]. For a gametocytocide of *P. falciparum*, a single dose of PQ (0.25 mg/kg) along with artemisinin-based combination treatment (ACT) without a requirement for G6PD deficiency testing has been recommended by the WHO [46–48].

## Conclusions

In summary, the presence of *G6PD Mahidol*^*G487A*^ and *PKLR*^R41Q^ was found in malaria patients in the Southeast Asia with MAFs of 7.1% and 2.5%, respectively. A deficiency in the enzyme G6PD by the *G6PD Mahidol*^*G487A*^ mutation exhibits a statistically significant correlation with haemolytic anaemia during malaria infection. Together, this study underlines the impact of host genetic background on haemolytic reactions and the benefit of screening for red cell enzymopathies and related mutations in patients before anti-malarial drug administration.

## Supplementary Information

**Additional file 1: Table S1.** Mean and standard deviation (SD) for clinical parameters of malaria patients without thalassaemia and haemoglobinopathies (*p*-values were determined using the Student’s *t*-test.)

## Data Availability

All data generated or analysed during this study are included in this published article.

## Abbreviations

G6PD: Glucose 6-phosphate dehydrogenase
PKLR: Pyruvate kinase
PQ: Primaquine
NADPH: Nicotinamide adenine dinucleotide phosphate
PCR–RFLP: Polymerase chain reaction–restriction fragment length polymorphism
MAF: Minor allele frequency
Hb: Haemoglobin
RBCs: Red blood cells
Hct: Haematocrit
MCV: Mean corpuscular volume
MCH: Mean corpuscular haemoglobin
MCHC: Mean corpuscular Hb concentration
RDW: Red cell distribution width
PKD: PK deficiency
IQR: Interquartile range
AMM: Adjusted male median
DOT: Directly observed treatment
ACT: Artemisinin-based combination treatment
