# Supplementary material for: Haematological profile of malaria patients with G6PD and PKLR variants (erythrocytic enzymopathies): a cross-sectional study in Thailand
Source: Malar J. 2022 Aug 30;21:250. doi: 10.1186/s12936-022-04267-7 (PMC9426002; doi:10.1186/s12936-022-04267-7)
Supplement: Supplementary file 1 — Additional file 1: Table S1. Mean and standard deviation (SD) for clinical parameters of malaria patients without thalassaemia and haemoglobinopathies (p-values were determined using the Student’s t-test.) [file 12936_2022_4267_MOESM1_ESM.docx]

| Clinical parameters  (n=126) | G6PD Phenotypic status (n= 126) | | | | | *G6PD* and *PKLR* mutations (n= 126) | | | | | | |
| --- | --- | --- | --- | --- | --- | --- | --- | --- | --- | --- | --- | --- |
|  | **G6PD normal**  **(n=98)** | **G6PD intermediate**  **(n=13)** | ***p*-value** | **G6PD deficiency**  **(n=15)** | ***p*-value** | **Wild-type**  **(n=107)** | **Mahidol**  **(n=12)** | ***p*-value** | **Other *G6PD* mutations**  **(n=2)** | ***p*-value** | ***PKLR*^R41Q^**  **(n=5)** | ***p*-value** |
| Hb  (g/dl) | 12.9±2.0 | 13.1±1.1 | 0.607 | 11.1±3.0 | **0.041** | 12.9±2.0 | 10.8±2.9 | **0.038** | 11.6±5.3 | 0.778 | 13.9±0.9 | 0.279 |
|  | (6.4-17.0) | (11.7-15.3) |  | (6.5-15.3) |  | (6.4-17.0) | (6.5-15.2) |  | (7.8-15.3) |  | (13.1-15.3) |  |
| RBCs  (x10^6^/ul) | 4.6±0.7 | 4.8±0.4 | 0.229 | 3.9±1.2 | 0.053 | 4.6±0.6 | 3.7±1.0 | **0.019** | 4.4±2.4 | 0.904 | 5.0±0.3 | 0.244 |
|  | (2.5-6.1) | (4.2-5.6) |  | (2.2-6.0) |  | (2.47-6.11) | (2.16-5.27) |  | (2.7-6.0) |  | (4.5-5.4) |  |
| Hct  (%) | 38.8±5.7 | 40.2±3.6 | 0.383 | 33.1±8.0 | **0.023** | 38.7±5.5 | 32.5±7.3 | **0.001** | 33.2±15.9 | 0.709 | 43.2±3.7 | 0.076 |
|  | (19.4-52.3) | (35.3-48.4) |  | (20.7-44.4) |  | (19.4-52.3) | (20.7-43.8) |  | (22.0-44.4) |  | (37.5-47.4) |  |
| MCV  (fl) | 83.8±5.9 | 83.5±5.5 | 0.832 | 85.5±7.2 | 0.351 | 83.6±5.9 | 87.2±6.8 | 0.056 | 77.9±6.2 | 0.174 | 87.2±2.6 | 0.184 |
|  | (64.1-96.5) | (69.1-88.8) |  | (71.4-95.9) |  | (64.1-96.5) | (71.4-95.9) |  | (73.5-82.2) |  | (83.7-90.0) |  |
| MCH  (pg/cell) | 28.1±2.5 | 27.2±1.5 | 0.223 | 28.3±2.5 | 0.763 | 28.0±2.4 | 28.7±2.6 | 0.394 | 27.2±2.7 | 0.646 | 28.1±0.8 | 0.938 |
|  | (17.7-32.2) | (23.2-29.3) |  | (23.2-30.7) |  | (17.7-32.2) | (2.2-30.7) |  | (25.3-29.1) |  | (27.3-29.3) |  |
| MCHC  (g/dl) | 33.2±3.0 | 32.7±1.3 | 0.531 | 30.9±8.3 | 0.318 | 33.2±2.9 | 29.9±9.2 | 0.269 | 35.0±0.6 | 0.387 | 32.2±1.8 | 0.475 |
|  | (9.0-36.0) | (30.9-34.80) |  | (3.0-36.6) |  | (9.0-36.0) | (3.0-36.6) |  | (34.5-35.4) |  | (30.3-35.0) |  |
| RDW  (%) | 14.8±1.3 | 14.8±0.7 | 0.940 | 14.7±1.4 | 0.902 | 14.8±1.3 | 14.8±1.4 | 0.957 | 14.4±2.2 | 0.637 | 15.1±1.6 | 0.577 |
|  | (12.9-19.1) | (13.4-16.2) |  | (12.3-17.5) |  | (12.9-19.1) | (12.3-17.5) |  | (12.8-15.9) |  | (13.1-16.9) |  |
| Reticulocyte  (%) | 1.6±1.1 | 1.2±0.3 | 0.219 | 3.4±0.5 | **0.036** | 1.6±1.1 | 3.7 | 0.063 | 3.0 | 0.212 | 1.6±1.2 | 0.941 |
|  | (0.6-7.4) | (0.5-1.6) |  | (0.3-7.8) |  | (0.5-7.4) | - |  | - |  | (0.7-3.8) |  |
| Platelet  (x10^3^/mm^3^) | 101.8±95.1 | 78.6±60.6 | 0.394 | 114.9±37.9 | 0.614 | 99.0±93.2 | 118.4±37.8 | 0.498 | 120.5±41.7 | 0.746 | 96.0±37.1 | 0.943 |
|  | (14.0-79.0) | (7.0-214.0) |  | (65.0-214.0) |  | (7.0-790.0) | (84.0-214.0) |  | (91.0-150.0) |  | (49.0-146.0) |  |
| MPV  (fl) | 17.9±75.4 | 10.5±2.0 | 0.725 | 9.8±1.4 | 0.693 | 17.3±72.1 | 9.8±1.5 | 0.731 | 10.8±0.1 | 0.899 | 9.0±1.0 | 0.799 |
|  | (7.2-738.0) | (7.8-13.6) |  | (8.4-13.5) |  | (7.2-738.0) | (8.4-13.5) |  | (10.7-10.8) |  | (7.7-10.5) |  |
| TB  (mg/dl) | 1.5±1.9 | 3.9±10.3 | 0.406 | 1.6±0.9 | 0.790 | 1.8±4.0 | 1.8±1.0 | 0.999 | 1.07±0.6 | 0.803 | 0.8±0.3 | 0.599 |
|  | (0.2-16.1) | (0.4-38.0) |  | (0.6-3.3) |  | (0.23-37.98) | (0.65-3.27) |  | (0.68-1.46) |  | (0.33-1.12) |  |
| DB  (mg/dl) | 0.7±1.6 | 2.9±9.0 | 0.401 | 0.7±0.6 | 0.997 | 1.0±3.5 | 0.8±0.7 | 0.844 | 0.5±0.2 | 0.843 | 0.4±0.1 | 0.675 |
|  | (0.0-12.8) | (0.2-33.0) |  | (0.1-2.4) |  | (0.0-32.97) | (0.09-2.35) |  | (0.4-0.7) |  | (0.1-0.5) |  |
| IDB  (mg/dl) | 0.7±0.5 | 1.0±1.4 | 0.513 | 0.9±0.6 | 0.351 | 0.8±0.7 | 1.0±0.6 | 0.335 | 0.6±0.4 | 0.649 | 0.5±0.2 | 0.342 |
|  | (0.1-3.4) | (0.2-5.0) |  | (0.3-2.5) |  | (0.13-5.01) | (0.38-2.45) |  | (0.3-0.8) |  | (0.2-0.7) |  |
| Parasitemia | 23049.5  ± 33677.8 | 31730.3  ± 63933.1 | 0.639 | 21706.8  ± 24023.5 | 0.886 | 24435.5  ± 38761.3 | 25728.18  ± 25703.6 | 0.914 | 10060.0  ± 2856.7 | 0.603 | 11598.0  ± 10020.7 | 0.463 |
| (parasite/ul) | (124-278250) | (1-185820) |  | (270-72750) |  | (1-278250) | (270-72750) |  | (8040-12080) |  | (1240-27050) |  |

**Bold**values indicate statistical significance at the *p*-value <0.05 level

**Additional file 1: Table S1.** Mean and standard deviation (SD) for clinical parameters of malaria patients without thalassaemia and haemoglobinopathies (*p*-values were determined using the Student’s *t*-test.)

A total of 129 patients with thalassaemia and haemoglobinopathies were excluded to study the associations between *G6PD* or *PKLR* mutations and haematological parameters. At the first visit prior to malaria treatment, 15 malaria patients with G6PD deficiency, compared to 98 malaria patients with normal G6PD activity levels, exhibited a significant decrease in haemoglobin levels (11.05 ± 3.04 g/dl *vs*. 12.93 ± 2.05 g/dl; *p* = 0.041). These patients also had a significant increase in reticulocyte count (3.40 ± 0.50% *vs*. 1.60 ± 1.10%; *p* = 0.036). Malaria patients with *G6PD Mahidol^G487A^* mutation (n=12) compared to wildtype patients without common Southeast Asian (SEA) mutations including the *G6PD Mahidol^G487A^* (n=107) exhibited a significant decrease in haemoglobin levels (10.77 ± 2.94 g/dl *vs*. 12.91 ± 1.98 g/dl; *p* = 0.038). These patients also had an increase of reticulocyte levels (3.70 % *vs.* 1.60 ± 1.10%; *p* = 0.063) (**Table 2**). There were no statistically differences in malaria patients with *PKLR^R41Q^* compared to those with wildtype.
